# Cannabis Legalization and Opioid Use Disorder in Veterans Health Administration Patients

**DOI:** 10.1001/jamahealthforum.2025.1369

**Published:** 2025-06-13

**Authors:** Zachary L. Mannes, Melanie M. Wall, Daniel M. Alschuler, Carol A. Malte, Mark Olfson, Ofir Livne, David S. Fink, Salomeh Keyhani, Katherine M. Keyes, Silvia S. Martins, Magdalena Cerdá, Dana L. Sacco, Sarah Gutkind, Charles C. Maynard, Scott Sherman, Andrew J. Saxon, Deborah S. Hasin

**Affiliations:** 1 Department of Emergency Medicine, Columbia University Irving Medical Center, New York, New York; 2 Department of Epidemiology, Columbia University Mailman School of Public Health, New York, New York; 3 New York State Psychiatric Institute, New York; 4 Health Services Research & Development Seattle Center of Innovation for Veteran-Centered and Value-Driven Care, Veterans Affairs (VA) Puget Sound Health Care System, Seattle, Washington; 5 Center of Excellence in Substance Addiction Treatment and Education, VA Puget Sound Health Care System, Seattle, Washington; 6 Department of Psychiatry, Columbia University Irving Medical Center, New York, New York; 7 San Francisco VA Medical Center, San Francisco, California; 8 Department of General Internal Medicine, University of California, San Francisco; 9 Department of Population Health, Center for Opioid Epidemiology and Policy, New York University Langone Health, New York; 10 Department of Population Health Sciences, Weill Cornell Medicine, New York, New York; 11 Department of Health Systems and Population Health, School of Public Health and Community Medicine, University of Washington, Seattle; 12 VA Manhattan Harbor Healthcare, New York, New York; 13 Department of Psychiatry & Behavioral Sciences, University of Washington School of Medicine, Seattle

## Abstract

**Importance:**

In the context of the US opioid crisis, factors associated with the prevalence of opioid use disorder (OUD) must be identified to aid prevention and treatment. State medical cannabis laws (MCL) and recreational cannabis laws (RCL) are potential factors associated with OUD prevalence.

**Objective:**

To examine changes in OUD prevalence associated with MCL and RCL enactment among veterans treated at the Veterans Health Administration (VHA) and whether associations differed by age or chronic pain.

**Design, Setting, and Participants:**

Using VHA electronic health records from January 2005 to December 2022, adjusted yearly prevalences of OUD were calculated, controlling for sociodemographic characteristics, receipt of prescription opioids, other substance use disorders, and time-varying state covariates. Staggered-adoption difference-in-difference analyses were used for estimates and 95% CIs for the relationship between MCL and RCL enactment and OUD prevalence. The study included VHA patients aged 18 to 75 years. The data were analyzed in December 2023.

**Main Outcome and Measures:**

*International Classification of Diseases, Ninth Revision, Clinical Modification* (*ICD-9-CM*) or *International Statistical Classification of Diseases, Tenth Revision, Clinical Modification* (*ICD-10-CM*) OUD diagnoses.

**Results:**

From 2005 to 2022, most patients were male (86.7.%-95.0%) and non-Hispanic White (70.3%-78.7%); the yearly mean age was 61.9 to 63.6 years (approximately 3.2 to 4.5 million patients per year). During the study period, OUD decreased from 1.12% to 1.06% in states without cannabis laws, increased from 1.13% to 1.19% in states that enacted MCL, and remained stable in states that also enacted RCL. OUD prevalence increased significantly by 0.06% (95% CI, 0.05%-0.06%) following MCL enactment and 0.07% (95% CI, 0.06%-0.08%) after RCL enactment. In patients aged 35 to 64 years and 65 to 75 years, MCL and RCL enactment was associated with increased OUD, with the greatest increase after RCL enactment among older adults (0.12%; 95% CI, 0.11%-0.13%). Patients with chronic pain had even larger increases in OUD following MCL (0.08%; 95% CI, 0.07%-0.09%) and RCL enactment (0.13%; 95% CI, 0.12%-0.15%). Consistent with overall findings, the largest increases in OUD occurred among patients with chronic pain aged 35 to 64 years following the enactment of MCL and RCL (0.09%; 95% CI, 0.07%-0.11%) and adults aged 65 to 75 years following RCL enactment (0.23%; 95% CI, 0.21%-0.25%).

**Conclusions and Relevance:**

The results of this cohort study suggest that MCL and RCL enactment was associated with greater OUD prevalence in VHA patients over time, with the greatest increases among middle-aged and older patients and those with chronic pain. The findings did not support state cannabis legalization as a means of reducing the burden of OUD during the ongoing opioid epidemic.

## Key Points

**Question:**

Were medical cannabis laws (MCLs) or recreational cannabis laws (RCLs) associated with opioid use disorder (OUD) in Veterans Health Administration patients?

**Findings:**

In this cohort study of Veterans Affairs Administration patients, from 2005 to 2022, OUD decreased from 1.12% to 1.06% in states without cannabis laws, increased from 1.13% to 1.19% in states that enacted MCL, and remained stable in states that also enacted RCL. MCL/RCL enactment was associated with greater OUD prevalence, with more substantial increases in OUD among middle-aged and older adults and those with chronic pain.

**Meaning:**

The study results suggest that MCL/RCL enactment was associated with increased OUD, suggesting that cannabis legalization is not an effective intervention to reduce the burden of OUD.

## Teaser

This cohort study examines changes in opioid use disorder prevalence associated with medical and recreational cannabis law enactment among veterans treated at the Veterans Health Administration and whether associations differed by age or chronic pain.

## Introduction

In the US, opioid use disorder (OUD) affects more than 6.5 million adults.^1^ OUD, which is characterized by a pattern of opioid use contributing to impairment and distress,^2^ is associated with opioid overdose^3,4,5^ and psychiatric, medical, and psychosocial health consequences.^1,6,7^ Many individual-level factors contribute to OUD risk, including younger age, male sex, opioid use, other substance use disorders (SUDs), and chronic pain persisting 3 months or longer.^8,9^ Unremitting rates of OUD^1^ make identifying OUD risk factors an ongoing, urgent public health need to aid in its prevention and treatment.

Cannabis legalization may be 1 factor associated with OUD prevalence. As of 2024, 39 US states have enacted medical cannabis laws (MCLs), and 24 states and Washington, DC, have additionally enacted recreational cannabis laws (RCLs). MCLs/RCLs could be associated with decreased OUD via 2 mechanisms. One involves pain management. While opioid prescriptions have declined,^1^ they are still widely dispensed for pain.^10,11^ Chronic pain is now among the most common medical conditions for which cannabis is authorized,^12^ and many adults perceive cannabis as an effective treatment for chronic pain.^13^ Ecological studies have indirectly suggested that cannabis is used in place of opioids following MCL enactment,^14,15,16,17,18,19,20^ potentially reducing opioid-related consequences, including development of OUD. The other mechanism involves cannabis as treatment for OUD, which is now a qualifying condition for medical cannabis in 3 US states (New York, New Jersey, and Pennsylvania).^21^ In some OUD treatment studies, cannabis use was associated with reductions in opioid dose, less opioid craving, and greater engagement and retention,^22,23^ potentially reducing OUD burden in states with legal access to cannabis. However, prospective studies have shown that cannabis use is associated with an increased risk of opioid use^24,25^ and may be used as a complement to opioids,^26^ which could be associated with increases in OUD if cannabis use is legalized.

Three studies have examined MCL enactment and OUD using data from the National Survey on Drug Use and Health,^27,28,29^ finding no associations between MCL and OUD. A recent study also found no association between RCL and OUD. However, these studies included adolescents, among whom OUD is rare,^30^ and used data before enactment of MCL or RCL in many states.

Veterans are disproportionately affected by chronic pain^31^ and vulnerable to health risks of cannabis use,^32^ and OUD has increased considerably in the Veterans Health Administration (VHA) since 2005.^33^ Therefore, using annual electronic health record data from the VHA, which is among the largest integrated health care systems in the US, we investigated trends in OUD from 2005 to 2022, assessing whether MCL/RCL enactment was associated with changes in OUD prevalence. We also examined MCL/RCL–associated changes in OUD by age because OUD rates vary with age^34^ and prior studies have shown that MCL/RCL are disproportionately associated with the risk of other SUD among older veterans.^35,36,37^ We further examined associations between MCL/RCL and OUD by chronic pain status, because cannabis legalization may also be associated with increased cannabis use as a substitute for opioids to treat pain, thereby reducing opioid use and the prevalence of OUD.

## Methods

### Sample and Procedure

Data from January 1, 2005, to December 31, 2022, were obtained from the VHA Corporate Data Warehouse, a database of patients receiving health care at VHA facilities. Data were organized into 18 annual cross-sectional datasets (N = 3 234 382-4 436 913). Veterans aged 18 to 75 years with at least 1 VHA health care encounter during a calendar year were included. We excluded patients in hospice/palliative care or those residing outside the 50 states and Washington, DC. We adhered to the Strengthening the Reporting of Observational Studies in Epidemiology (STROBE) reporting guidelines. The institutional review boards at the VAs Puget Sound and New York Harbor Healthcare Systems and New York State Psychiatric Institute approved this study. Waivers/exemptions of informed consent were granted by New York State Psychiatric Institute, VA Puget Sound, and VA New York Harbor Healthcare Systems institutional review boards.

### Outcome

Patients with OUD were aggregated yearly from 2005 to 2022. OUD was identified using the *International Classification of Diseases, Ninth Revision, Clinical Modification (ICD-9-CM)* (304.0x, 304.7x, and 305.5x) from January 1, 2005, to September 30, 2015, and *International Statistical Classification of Diseases, Tenth Revision, Clinical Modification (ICD-10-CM)* (F11.1X, F11.2X) from October 1, 2015, to December 31, 2022. Patients were considered positive for OUD if they received 1 or more OUD diagnoses during an outpatient or inpatient encounter at any VA health care facility each year. Codes for OUD in remission were excluded (*ICD-9-CM*: 304.03X, 304.73, 305.53X; *ICD-10-CM*: F11.11, F11.21).

### Exposure

State-year variables were created to indicate MCL and/or RCL enactment, meaning an operational law in which people had legal cannabis access. Patients’ state of residence was identified by the location of their last VHA health care encounter each year. States were categorized yearly as no MCL/RCL, MCL, and MCL/RCL. From 2005 to 2022, 37 states and Washington, DC, enacted MCL or RCL, including 17 states that enacted only MCL, 12 states and Washington, DC, that enacted MCL and RCL, and 8 states that had MCL before 2005 and enacted RCL during the study period. All states with RCL had previously enacted an MCL.

### Covariates

We included covariates associated with OUD.^9^ Demographic characteristics included sex (female, male), age (<35, 35-64, 65-75 years), and self-reported race and ethnicity (Hispanic, non-Hispanic Black, non-Hispanic White, other race [Asian, American Indian/Alaskan Native, or Pacific Islander/Native Hawaiian] or multiracial, or unknown). We also included time-varying yearly state variables from the American Community Survey data, including state percentages of male individuals, self-reported race and ethnicity (Hispanic, non-Hispanic Black, or non-Hispanic White), age 18 years or older, unemployment, an income less than the federal poverty level, and median household income using R tidycensus (R Foundation). We used 1-year percentages for years 2005 to 2008 and 5-year percentages from 2009 to 2022.^38^ Consistent with previous articles,^35,36^ we included *ICD-9-CM* or *ICD-10-CM* codes to assess chronic pain conditions (yes, no). We also included *ICD* codes for other substance use disorders (yes, no; eAppendix in Supplement 1). We required 2 or more outpatient or 1 or more inpatient pain diagnoses and 1 outpatient or inpatient diagnosis within each study year for pain and SUD, respectively. Measures of 30-day or longer receipt of prescription opioids (yes, no) and state enactment of a prescription drug monitoring program (PDMP) mandatory access law (requiring that clinicians review the PDMP before prescribing opioids)^39,40^ were also examined.

### Statistical Analysis

OUD prevalences were aggregated annually, overall, and as a function of cannabis legalization status (MCL, RCL, or no cannabis law). We calculated yearly adjusted OUD prevalence using linear binomial regression models, including year and an interaction term for MCL/RCL status year, which were adjusted for sex, race and ethnicity, continuous age, SUD, receipt of prescription opioids, PDMP mandatory access law, and state-level covariates. We then reran models stratified by age groups (<35, 35-64, or 65-75 years)^41,42^ and chronic pain status (eMethods in Supplement 1).^35,36^

The associations between cannabis legalization and OUD were assessed using staggered-adoption difference-in-difference (DiD) estimates, overall and stratified by age groups controlling for fixed state and time effects. The DiD model used MCL/RCL–enacted states as independent controls, comparing years after MCL/RCL enactment with years before enactment, accounting for OUD trends from no MCL/RCL states during the same period. For each state-year, a time-varying measure was created to indicate MCL/RCL status. DiD effects were estimated as the change in OUD associated with state transition from no cannabis laws to MCL and from MCL to MCL/RCL, using comparator data from the 13 no cannabis law states and the 17 MCL states that did not enact RCL by 2022. DiD estimates and 95% CIs were derived by fitting a linear binomial regression model at the individual level that included state fixed effects, year, time-varying MCL/RCL status, individual demographic characteristics (including SUD), receipt of prescription opioids, PDMP mandatory access laws, and state-level covariates. The resulting DiD estimates indicated the association with OUD prevalence of a state moving from no MCL/RCL to MCL or from MCL to RCL. Preenactment OUD trends were examined and found to be consistent with the parallel trends assumption of DiD analyses (eMethods in Supplement 1).

We also conducted 2 sets of sensitivity analyses using an approach similar to main analyses. We examined the association between legalized dispensaries and OUD by replacing our MCL/RCL variables with state-year variables indicating years that legally protected dispensaries were operational for medical cannabis in MCL states and for recreational cannabis also enacting RCL,^43^ as well as the lagged effects of cannabis legalization by replacing MCL/RCL state/year variables with 1-year postenactment dates, overall and by chronic pain status.

## Results

### Sample Characteristics

From 2005 to 2022, most patients were male (86.7.%-95.0%) and non-Hispanic White (70.3%-78.7%); the yearly mean age was 61.9 to 63.6 years. The prevalence of younger veterans (aged <35 years), women, and patients of racial and ethnic minority groups increased during the study period (eTable 3 in Supplement 1).

### Overall and Age-Stratified OUD Prevalence by MCL/RCL Status

**Figure 1.**
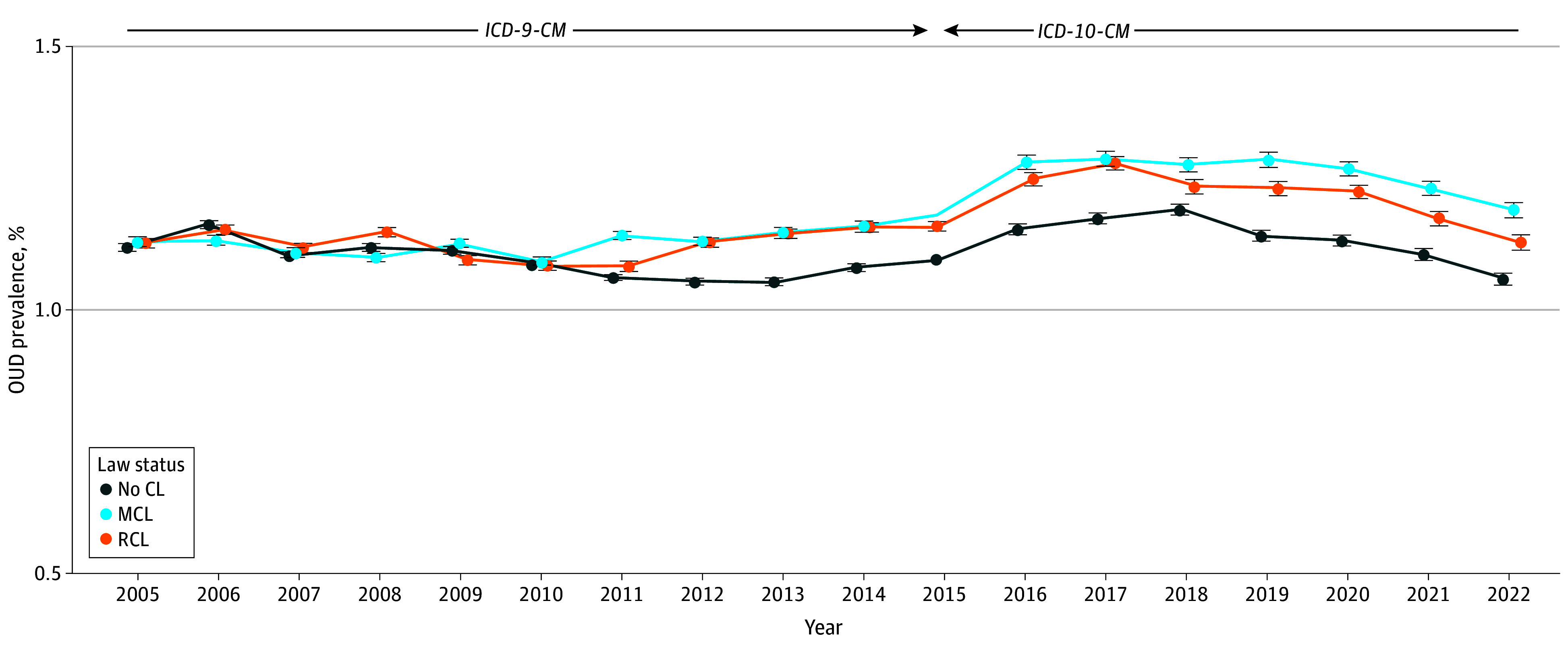
Trends in Prevalence of Opioid Use Disorder (OUD) Among Veterans Health Administration Patients From 2005 to 2022 by Medical Cannabis Law (MCL)/Recreational Cannabis Law (RCL) Status Overall trend in OUD as a function of cannabis law status from 2005 to 2022. In 2015, the predicted diagnostic prevalence of OUD is an aggregate across some patients with a diagnosis coded with *International Classification of Diseases, Ninth Revision, Clinical Modification (ICD-9-CM)* and others with *International Statistical Classification of Diseases, Tenth Revision, Clinical Modification (ICD-10-CM)* due to the change in *ICD* coding midyear. Estimates were adjusted for age, sex, race and ethnicity, mandatory prescription drug monitoring program access law, receipt of 30 days or longer of prescription opioids, other substance use disorder (alcohol use disorder, cocaine use disorder, stimulant use disorder, sedative use disorder, hallucinogen related disorders, inhalant-related disorders, or other psychoactive substance–related disorder), and time-varying state covariates, including yearly state-level median income and yearly state rates of male individuals, Black individuals, Hispanic individuals, White individuals, those in the poverty category, those 18 years and older, and those who are unemployed. Error bars indicate 95% CIs. CL indicates cannabis law.

The adjusted overall prevalence of OUD remained stable from 1.12% in 2005 to 1.12% in 2022, peaking at 1.25% in 2017 (eTable 4 in Supplement 1). From 2005 to 2022, OUD prevalence decreased by 0.06% in no cannabis law states, increased by 0.06% in MCL states, and remained stable in states with additional RCLs (Figure 1; Table 1). Yearly age-stratified prevalences of OUD by MCL/RCL status are shown in eTable 5 in Supplement 1. From 2005 to 2022, in no cannabis law states, OUD increased by 0.07% in patients aged 35 to 64 years and 0.16% in those aged 65 to 75 years and declined by 0.07% in patients younger than 35 years. In MCL and RCL states, OUD increased in patients aged 65 to 75 years (MCL, 0.27%; RCL, 0.41%) but decreased in patients younger than 35 years (MCL, −0.25%; RCL, −0.21%) and aged 35 to 64 years (MCL, −0.02%; RCL, −0.46%).

**Table 1.** Adjusted Opioid Use Disorder (OUD) Prevalence in Veterans Health Administration Patients in 2005 and 2022 by Medical Cannabis Law (MCL)/Recreational Cannabis Law (RCL) Status Overall and by Age Group

Type of state	Overall			Age 18-34 y			Age 35-64 y			Age 65-75 y		
	OUD prevalence^a^		Absolute change, %	OUD prevalence^b^		Absolute change, %	OUD prevalence^b^		Absolute change, %	OUD prevalence^b^		Absolute change, %
	2005	2022		2005	2022		2005	2022		2005	2019	
No. of patients	3 234 382	4 436 883	NA	182 809	468 729	NA	1 993 492	2 338 600	NA	1 058 081	1 629 554	NA
No CL^c^	1.12	1.06	−0.06	1.54	1.47	−0.07	0.49	0.56	0.08	0.45	0.61	0.16
MCL^d^	1.13	1.19	0.06	1.71	1.46	−0.25	0.73	0.71	−0.02	0.45	0.72	0.27

Abbreviations: CL, cannabis law; NA, not applicable.

^a^ Adjusted for categorical age, sex, race and ethnicity, age × race and ethnicity × sex interactions, mandatory prescription drug monitoring program access law, receipt of 30 days or more of prescription opioids, other substance use disorder (alcohol use disorder, cocaine use disorder, stimulant use disorder, sedative use disorder, hallucinogen related disorders, inhalant-related disorders, or other psychoactive substance–related disorder), and time-varying state covariates, including yearly state-level median income, yearly state rates of male individuals, Hispanic individuals, non-Hispanic Black individuals, non-Hispanic White individuals, those with incomes less than the federal poverty level, those 18 or older, and those who are unemployed.

^b^ Adjusted for continuous age as well as the variables outlined in footnote a.

^c^ Thirteen states.

^d^ Seventeen states.

### Overall and Age-Stratified Associations of MCL/RCL Enactment and OUD Prevalence

In the overall sample, DiD results indicated a 0.06% (95% CI, 0.05%-0.06%; *P* < .001) increase in OUD prevalence following MCL enactment and a 0.07% (95% CI, 0.06-0.08; *P* < .001) increase after RCL enactment. In patients aged 35 to 64 years, MCL enactment was associated with a 0.05% (95% CI, 0.04%-0.06%; *P* < .001) increase in OUD prevalence and 0.04% (95% CI, 0.02%-0.05%; *P* < .001) increase in OUD after RCL enactment. These results were consistent in the group aged 65 to 75 years, for whom MCL and RCL enactment were associated with a 0.04% (95% CI, 0.03%-0.04%; *P* < .001) and 0.12% (95% CI, 0.11%-0.13%; *P* < .001) increase in OUD, respectively. Cannabis law enactment was not associated with OUD in the group aged 18 to 34 years (Table 2).

**Table 2.** State Medical Cannabis Law (MCL) and Recreational Cannabis Law (RCL) Enactment and Opioid Use Disorder Prevalence in Veterans Health Administration Patients

Change in state law^a^	Overall		Age 18-34 y		Age 35-64 y		Age 65-75 y	
	DiD law result (95% CI)^b^	*P* value	DiD law result (95% CI)^c^	*P* value	DiD law result (95% CI)^c^	*P* value	DiD law result (95% CI)^c^	*P* value
No CL to MCL	0.06 (0.05 to 0.06)	<.001	0.014 (−0.004 to 0.032)	.12	0.050 (0.041 to 0.060)	<.001	0.036 (0.029 to 0.042)	<.001
MCL to RCL	0.07 (0.06 to 0.08)	<.001	−0.018 (−0.039 to 0.003)	.10	0.035 (0.022 to 0.048)	<.001	0.119 (0.108 to 0.130)	<.001

Abbreviations: CL, cannabis law; DiD, difference-in-difference.

^a^ From 2005 to 2022, 26 states and Washington, DC, enacted MCL only from 2005 to 2022 and 11 states and Washington, DC, transitioned from MCL only to RCL/MCL. Three states and Washington, DC, made both changes between 2005 and 2022 (ie, no CL to MCL only and then later to RCL/MCL), and therefore contributed data to both associations. There were 15 states (2 with MCLs only and 13 with no CLs in 2022) that made no law changes between 2005 and 2022; in the DiD model, they contributed to background secular trends. Model estimated effects represented the absolute increase or decrease in opioid use disorder prevalence associated with law enactment. The DiD model compared the years after enactment (up to 2022 or until the next law change) in each state to the years before enactment (since 2005 or the previous law change) in the same state and controls for contemporaneous trends in other states that have not yet passed the respective law.

^b^ Percentage change in prevalence of opioid use disorder. Adjusted for categorical age, sex, race and ethnicity, mandatory prescription drug monitoring program access law, receipt of 30 days or more of prescription opioids, other substance use disorder (alcohol use disorder, cocaine use disorder, stimulant use disorder, sedative use disorder, hallucinogen-related disorders, inhalant-related disorders, or other psychoactive substance–related disorder), and time-varying state covariates, including yearly state-level median income and yearly state rates of male individuals, Hispanic individuals, non-Hispanic Black individuals, non-Hispanic White individuals, those in the poverty category, those 18 years and older, and those who are unemployed.

^c^ Adjusted for continuous age and the variables described in footnote b.

### State-Specific Associations of MCL/RCL Enactment and OUD

**Figure 2.**
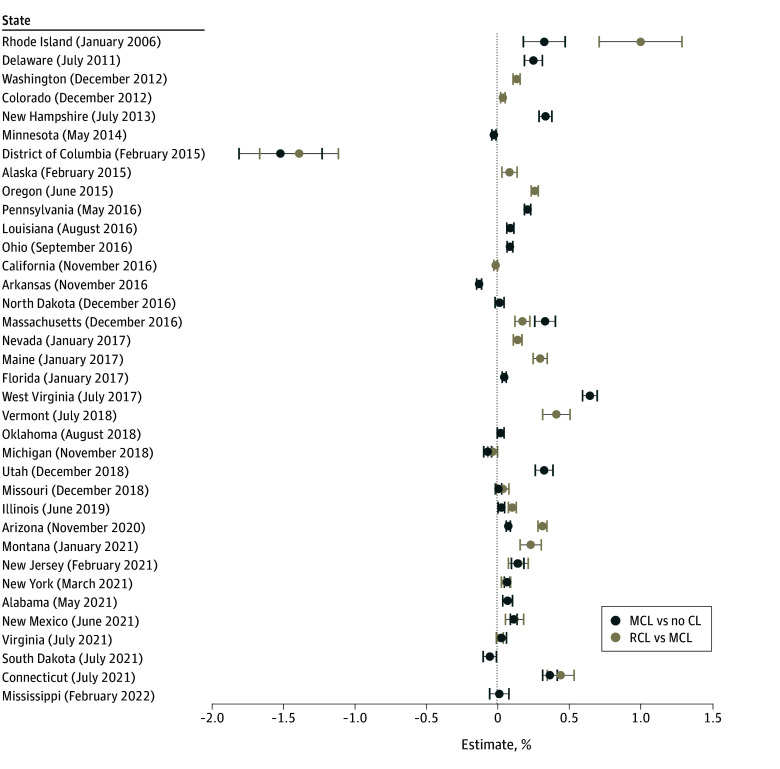
State-Specific Associations of Medical Cannabis Law (MCL) and Recreational Cannabis Law (RCL) Enactment With Opioid Use Disorder in Veterans Health Administration Patients by the Most Recent Month per Year of Cannabis MCL or RCL Enactment Estimates adjusted for age, sex, race and ethnicity, mandatory prescription drug monitoring program access law, receipt of 30 days or longer of prescription opioids, other substance use disorder (alcohol use disorder, cocaine use disorder, stimulant use disorder, sedative use disorder, hallucinogen related disorders, inhalant-related disorders, or other psychoactive substance–related disorder), and time-varying state covariates, including yearly state-level median income and yearly state rates of male individuals, Black individuals, Hispanic individuals, White individuals, those in the poverty category, those 18 years and older, and those who are unemployed. In states that changed to MCL and RCL during the period, the MCL/RCL association plotted was compared with no cannabis law (CL) for comparison with the MCL only vs no CL association. Point estimates and 95% CIs from the staggered-adoption difference-in-difference (DiD) regression models are displayed. From 2005 to 2022, 26 states and Washington, DC, enacted MCL only from 2005 to 2022 and 11 states and Washington, DC, transitioned from MCL only to RCL/MCL. Three states and Washington, DC, made both changes between 2005 and 2022 (ie, no CL to MCL only and then later to RCL/MCL) and therefore contributed data to both associations. There were 15 states (2 with MCLs only and 13 with no CLs in 2022) that made no law changes between 2005 and 2022; in the DiD model, they contributed to background secular trends. Model estimated effects represent the absolute increase or decrease in OUD prevalence associated with law enactment. The DiD model compared the years after enactment (up to 2022 or until the next law change) in each state to the years before enactment (since 2005 or the previous law change) in the same state and controls for contemporaneous trends in other states that have not yet passed the respective law.

Of the 17 states that only enacted MCL, 9 states demonstrated a significant increase in OUD, 1 state had a significant decline in OUD (Arizona), and 7 states showed no significant change in OUD. In the 20 states that also passed RCL, enactment was associated with increased OUD prevalence in 17 states. Washington, DC, was the only jurisdiction where RCL enactment was associated with a decline in OUD (Figure 2).

Compared with patients without chronic pain (approximately 1.2-2.5 million patients per year), those with chronic pain (approximately 1.9-2.0 million patients per year) were disproportionately affected by OUD following MCL/RCL enactment (eTable 6 in Supplement 1). Among patients with chronic pain, MCL and RCL enactment were associated with a 0.08% (95% CI, 0.07%-0.09%; *P* < .001) and 0.13% (95% CI, 0.12%-0.15%; *P* < .001) increase in OUD prevalence, respectively, while patients without chronic pain had only a 0.03% (95% CI, 0.02%-0.03%; *P* < .001) and a 0.01% (95% CI, 0.01%-0.02%; *P* = .03) increase in OUD following MCL and RCL enactment, respectively. In patients aged 35 to 64 years with chronic pain, MCL enactment and RCL enactment was associated with a 0.09% (95% CI, 0.07%-0.11%; *P* < .001) increase in OUD (eTable 7 in Supplement 1). Cannabis legalization was also associated with increased OUD in patients aged 65 to 75 years with chronic pain, an increase of 0.06% (95% CI, 0.04%-0.07%; *P* < .001) in MCL states and 0.23% (95% CI, 0.21%-0.25%; *P* < .001) in RCL states. OUD also increased in patients aged 65 to 75 years without chronic pain, but to a lesser degree, with an increase of 0.02% (95% CI, 0.01%-0.02%; *P* < .001) after MCL enactment and 0.03% (95% CI, 0.02%-0.04%; *P* < .001) following RCL enactment. In patients aged 18 to 34 years with chronic pain, MCL enactment was associated with a 0.07% increase in OUD (95% CI, 0.04%-0.10%; *P* < .001), with no significant change in OUD following RCL enactment.

### Sensitivity Analyses

Findings from sensitivity analyses were generally consistent with the main results (eTables 8-10 in Supplement 1). Enactment of operational medical dispensaries was associated with 0.07% (95% CI, 0.06%-0.08%; *P* < .001) increase in OUD compared with states without medical dispensaries, while operational recreational dispensaries further increased OUD by 0.05% (95% CI, 0.04%-0.06%; *P* < .001) compared with states with only medical dispensaries (eTable 8 in Supplement 1). Following the operationalization of medical cannabis dispensaries, OUD increases were greatest in the group aged 35 to 64 years, particularly those with chronic pain (DiD, 0.12%; 95% CI, 0.10%-0.13%; *P* < .001), while patients aged 65 to 75 years with chronic pain (DiD, 0.20%; 95% CI, 0.17%-0.22%; *P* < .001) were most affected by the operationalization of recreational dispensaries (eTable 9 in Supplement 1). Using 1-year postenactment lags did not significantly change the results (eTables 10 and 11 in Supplement 1).

## Discussion

In this cohort study, we examined changes in the yearly prevalence of OUD that was associated with MCL and/or RCL enactment among VHA patients from 2005 to 2022. MCL enactment was associated with small, yet significant increases in OUD overall and in middle-aged and older-aged adults. RCL enactment was associated with further increases in OUD, particularly among older adults with chronic pain. These results were similar in sensitivity analyses examining operational cannabis dispensaries and 1-year lags. Our findings did not support MCL or RCL enactment as a means of reducing the burden of OUD. Instead, MCLs/RCLs could potentially be associated with an increased OUD risk among VHA patients during the ongoing opioid epidemic. These are timely considerations given that the US government has considered cannabis policy reform for the VHA.^44,45^

There are several possible explanations of our findings. Cannabis use may be associated with an increased risk of consuming other substances, either by providing more opportunities through access to the same illicit markets or peers who use drugs, or by lowering the threshold for addiction to other substances, including opioids.^46^ Clinical and epidemiological studies have also shown increasingly prevalent patterns of course, in which individuals who use cannabis are more likely to use opioids.^26,47^ This course may stem from shared risk factors or the use of cannabis to enhance the effects of opioids. Several prospective studies have demonstrated that cannabis use is associated with an increased risk of opioid use and may be used with opioids, for instance, to augment relief of pain,^25,26,48,49^ a condition which was prevalent in our sample. A 25-year longitudinal study found a strong prospective association between use of cannabis and subsequent use of opioids, with the likelihood increasing as cannabis use frequency increased.^48^ Similar findings were observed in another prospective study of adults, in which nonmedical opioid use was more likely to occur in those with vs without cannabis use, with increasing risk among those with pain.^24^ OUD is more likely to develop in individuals using cannabis, particularly among those with frequent cannabis use,^24^ which has become substantially more prevalent in the US in recent years.^50^ Our study suggests that as access to cannabis increases with MCL/RCL, individuals may be more likely to experience these risks.

Our findings were consistent with 3 national studies that showed that MCL was not associated with a reduced prevalence of OUD,^27,28,29^ with adults 50 years or older demonstrating the greatest increase in OUD following MCL enactment.^28^ Some ecological studies have suggested that cannabis legalization is associated with reductions in opioid use,^14^ a necessary although not sufficient condition for the development of OUD. However, a study of national clinical data found no MCL-related reductions in opioid use among patients with chronic pain.^51^ In our study, VHA patients with chronic pain (>50% of patients)^36^ demonstrated increased OUD following MCL enactment. While medical cannabis has been authorized for chronic pain, evidence for its efficacy as a pain treatment remains inconclusive.^52^ Therefore, adults unable to manage their chronic pain with cannabis may transition to use of prescribed or nonprescribed opioids or augment the effects of opioids with cannabis,^26^ putting them at risk for OUD. Older adults are particularly vulnerable to the adverse effects of cannabis due to their lower substance use tolerance,^53,54^ increasing their risk of developing OUD if cannabis serves as a gateway to opioid use. This could be especially salient among older VHA patients, who may not have used cannabis during their earlier adult years when federal and military policy prohibited use, but whose willingness to use cannabis may have increased with exposure to marketing for cannabis as a medical treatment.^55,56^

RCL enactment was also associated with increases in OUD prevalence overall and in most individual states. These findings contrast with a recent study that showed that RCL enactment was not associated with OUD^29^ in a sample that was predominantly younger than 35 years. However, the age distribution of our sample was markedly different and comprised of mostly middle-aged and older adults. When we stratified by age group, RCL was not associated with OUD in younger patients (age 18-34 years) with or without chronic pain, as cannabis use was already prevalent in this age group before enactment of cannabis laws, and the incremental effects of RCL in this group may have been minimal.^34^ However, RCL enactment was associated increased OUD in middle-aged (age 35-64 years) and older adults (age 65-75 years), particularly in older adults. Older patients may have been naive to the highly potent forms of cannabis that became available after RCL enactment,^55^ and increased recreational cannabis use following RCL may have served as a risk factor to opioid use and OUD, especially in older adults with chronic pain. Future research is needed to understand if these associations extend to these age groups in the general population, which will have implications for tailored OUD prevention strategies.

### Limitations

This study had limitations. VHA veterans are mostly male, White, and older, with lower financial resources and high rates of medical comorbidities.^57^ Therefore, findings may not be generalizable, although they also suggest that MCL/RCL enactment disproportionately affects OUD in older patients and those with chronic pain, 2 groups that are increasing in the general population.^58^ Second, medical conditions designated with *ICD* codes may be underdiagnosed, and OUD may be underestimated or misdiagnosed in health record or claims data, including in the VHA.^59,60,61^ However, we limited OUD misclassification by excluding OUD cases in remission, and OUD rates in our sample were similar to National Survey on Drug Use and Health rates, a nationally representative data source.^62^ Third, the *ICD-9-CM* to *ICD-10-CM* transition may have affected clinical estimates of OUD. However, our prevalences of OUD resembled those observed in the general population and were consistent with non-VHA data demonstrating that OUD peaked around 2016 to 2017.^1^ Our DiD estimates accounted for the transition from *ICD-9* to *ICD-10* by using states with no MCL/RCL as contemporaneous secular controls. Therefore, this increase in OUD likely did not meaningfully affect our results. Fourth, the VHA health record lacks information on cannabis use, so we were unable to assess frequency of use, route of administration, or cannabis potency in associated with OUD. Fifth, we did not collect data on patient disability or changes to VHA health care eligibility due to cannabis legalization. These are important areas for future research, especially given increasing cannabis potency and the risk of psychiatric and SUDs and potential disability due to these health conditons.^63^

## Conclusions

This cohort study demonstrated that MCL was associated with increased rates of OUD, with even greater increases in middle-aged and older adults, and patients with chronic pain who were residing in states that also enacted RCL. Findings suggest that cannabis policies and increasing access to legal cannabis were not associated with a reduced burden of OUD. US states considering cannabis legislation to combat the opioid epidemic should consider potential unintended consequences on the prevalence of OUD and other opioid-related health outcomes.^64^ Our results should encourage careful regulation of cannabis distribution following legalization and monitoring of OUD symptoms among patients residing in MCL/RCL states, alongside concerted surveillance efforts in older adults who may be particularly susceptible to cannabis and opioid use risks. Because our study was among the first to our knowledge to examine associations of MCL/RCL with OUD, general population studies are needed to assess the effect of these laws on OUD and other opioid-related harms, including opioid overdose. Moreover, future studies should examine whether certain clinical populations are susceptible to OUD following MCL/RCL enactment, including patients with psychiatric disorders or those with specific pain conditions, or whether cannabis laws may reduce substance use–related stigma or increase substance use reporting and treatment utilization and whether these factors could partially account for changes in OUD following enactment of MCL/RCL. These studies will help identify the mechanisms of the associations we found, inform clinical policies associated with cannabis use, and guide future treatment and prevention efforts of OUD during a time when rates of OUD and opioid overdoses remain high.^1,5^

## Notes

Supplement 1. eMethods. The parallel trends assumption in difference-in-difference estimation for cannabis law state policy eTable 1. Event times used in diff-in-diff estimation for each state for MCL eTable 2. Event times used in diff-in-diff estimation for each state for RCL eFigure 1. Staggered adoption of when MCL and RCL were passed in all 50 states and Washington D.C.: 2005 to 2022 eFigure 2. Event study for OUD among VHA patients eTable 3. Sample demographics by opioid use disorder status: 2005 and 2022 eTable 4. Yearly overall prevalence of opioid use disorder eTable 5. Yearly opioid use disorder prevalence by state cannabis law status, overall and by age groups eTable 6. Adjusted OUD prevalence in Veterans Health Administration patients with versus without chronic pain in 2005 and 2022, by MCL/RCL, by age group eTable 7. State MCL and RCL enactment and opioid use disorder prevalence in Veterans Health Administration patients with versus without chronic pain, overall and by age group eTable 8. State dispensary enactment and opioid use disorder prevalence in Veterans Health Administration patients, overall and by age group eTable 9. State dispensary status and opioid use disorder prevalence in Veterans Health Administration patients with versus without chronic pain, overall and by age group eTable 10. State 1-year lag of MCL and RCL enactment and opioid use disorder prevalence in Veterans Health Administration patients, overall and by age group eTable 11. State 1-year lag MCL and RCL enactment and opioid use disorder prevalence in Veterans Health Administration patients with versus without chronic pain, overall and by age group eAppendix. *ICD-9-CM* and *ICD-10-CM* codes for substance use disorders

Supplement 2. Data sharing statement
