# Supplementary material for: Cannabis Legalization and Opioid Use Disorder in Veterans Health Administration Patients
Source: JAMA Health Forum. 2025 Jun 13;6(6):e251369. doi: 10.1001/jamahealthforum.2025.1369 (PMC12166489; doi:10.1001/jamahealthforum.2025.1369)
Supplement: Supplement 1. — eMethods. The parallel trends assumption in difference-in-difference estimation for cannabis law state policy eTable 1. Event times used in diff-in-diff estimation for each state for MCL eTable 2. Event times used in diff-in-diff estimation for each state for RCL eFigure 1. Staggered adoption of when MCL and RCL were passed in all 50 states and Washington D.C.: 2005 to 2022 eFigure 2. Event study for OUD among VHA patients eTable 3. Sample demographics by opioid use disorder status: 2005 and 2022 eTable 4. Yearly overall prevalence of opioid use disorder eTable 5. Yearly opioid use disorder prevalence by state cannabis law status, overall and by age groups eTable 6. Adjusted OUD prevalence in Veterans Health Administration patients with versus without chronic pain in 2005 and 2022, by MCL/RCL, by age group eTable 7. State MCL and RCL enactment and opioid use disorder prevalence in Veterans Health Administration patients with versus without chronic pain, overall and by age group eTable 8. State dispensary enactment and opioid use disorder prevalence in Veterans Health Administration patients, overall and by age group eTable 9. State dispensary status and opioid use disorder prevalence in Veterans Health Administration patients with versus without chronic pain, overall and by age group eTable 10. State 1-year lag of MCL and RCL enactment and opioid use disorder prevalence in Veterans Health Administration patients, overall and by age group eTable 11. State 1-year lag MCL and RCL enactment and opioid use disorder prevalence in Veterans Health Administration patients with versus without chronic pain, overall and by age group eAppendix. ICD-9-CM and ICD-10-CM codes for substance use disorders [file jamahealthforum-e251369-s001.pdf]

Supplemental Online Content

Mannes ZL, Wall MM, Alschuler DM, et al. Cannabis legalization and opioid use disorder in Veterans Health Administration patients. *JAMA Health Forum*. 2025;6(6):e251369. doi:10.1001/jamahealthforum.2025.1369

eMethods. The parallel trends assumption in difference-in-difference estimation for cannabis law state policy
eTable 1. Event times used in diff-in-diff estimation for each state for MCL
eTable 2. Event times used in diff-in-diff estimation for each state for RCL
eFigure 1. Staggered adoption of when MCL and RCL were passed in all 50 states and Washington D.C.: 2005 to 2022
eFigure 2. Event study for OUD among VHA patients
eTable 3. Sample demographics by opioid use disorder status: 2005 and 2022
eTable 4. Yearly overall prevalence of opioid use disorder
eTable 5. Yearly opioid use disorder prevalence by state cannabis law status, overall and by age groups
eTable 6. Adjusted OUD prevalence in Veterans Health Administration patients with versus without chronic pain in 2005 and 2022, by MCL/RCL, by age group
eTable 7. State MCL and RCL enactment and opioid use disorder prevalence in Veterans Health Administration patients with versus without chronic pain, overall and by age group
eTable 8. State dispensary enactment and opioid use disorder prevalence in Veterans Health Administration patients, overall and by age group
eTable 9. State dispensary status and opioid use disorder prevalence in Veterans Health Administration patients with versus without chronic pain, overall and by age group
eTable 10. State 1-year lag of MCL and RCL enactment and opioid use disorder prevalence in Veterans Health Administration patients, overall and by age group
eTable 11. State 1-year lag MCL and RCL enactment and opioid use disorder prevalence in Veterans Health Administration patients with versus without chronic pain, overall and by age group
eAppendix. *ICD-9-CM* and *ICD-10-CM* codes for substance use disorders

This supplemental material has been provided by the authors to give readers additional information about their work.

© 2025 Mannes ZL et al. *JAMA Health Forum*.

eMethods. The parallel trends assumption in difference-in-difference estimation for cannabis law state policy

Difference-in-Difference (DiD) analyses estimate the effect of a specific intervention or treatment (e.g., passage of a law or enactment of a policy) by comparing the changes in outcomes over time between groups exposed and unexposed to the intervention. The parallel trends assumption is often used in interpreting the results of DiD analyses. As applied to this study, the assumption is that the trends found in states that enacted medical or recreational cannabis laws (MCL or RCL) would have been the same (i.e. parallel) as the trends in states that did not pass laws if the enacting states had not, in fact, passed the laws (i.e. the counterfactual). The parallel trends assumption is what allows the differencing (in the difference-in-difference estimation) to approximate a causal effect, and in practice, the parallel trends assumption is often made so that difference-in-difference estimates can be considered *causal* effect estimates.

We conservatively do not draw causal conclusions from our difference-in-difference estimates of MCL and RCL effects in this observational study, preferring to emphasize the estimates as descriptive of the changes in OUD. However, understanding what would be needed for these estimates to provide evidence of a causal effect of the policies is useful.

The parallel trends assumption is not directly testable since the counterfactual condition does not exist. However, a common way to assess the assumption is to examine the trends in the states prior to enacting MCL or RCL to the states that do not enact MCL or RCL. If the trends in states that eventually enact MCL or RCL are the same prior to enactment as the trends in states that do not enact MCL or RCL, then perhaps they would have continued to be similar post-enactment if the laws had not changed and inferring that a causal effect could be reasonable. However, alternatively, if rates had already begun to trend upwards in MCL or RCL states *prior* to MCL or RCL enactment, then attributing a causal effect of MCL or RCL on rates is less reasonable. This assessment of the parallel trend in the pre-treatment period is not without problems[1] and does not conclusively test the untestable parallel trends assumption. Nevertheless, the method is common way to assess the reasonableness of conclusions from DiD analyses. In the present case, assessing pre-policy trends in the staggered-adoption scenario (where states enact laws at different times) creates an additional difficulty since the pre-enactment time periods all differ across states.

eTables 1 and 2 below show the states that enacted MCL and RCL during the period from 2005 to 2022 and their "event times" observed during this period (i.e. how many years they were observed prior to and after enactment with zero being the year of passage). The parallel trends prior to enactment can then be assessed by showing that the expected difference in OUD prevalence for these states (in aggregate) for each year prior to enactment is not different than the respective OUD prevalence in the reference group (i.e., the states that did not enact MCL or RCL up to 2022).

Because of the staggered adoption of cannabis laws (eFigure 1) and varying number of states at each event time (eTables 1 and 2), we use the Sun & Abraham estimator[2] implemented in the R package fixest.[3] Demographic covariates included in the model were the same as those used in the primary analyses, i.e. individual-level age group, sex, and race and ethnicity, and state-level census variables. When estimating the RCL event time differences we further controlled for a time-varying indicator of whether MCL was enacted or not. One limitation of this software is its use of the linear rather than binomial link function for the outcome, which in our case is dichotomous OUD (yes/no). Because of this, we avoid strict testing conclusions. However, as can be seen in eFigure 2, the difference between OUD in the law-enacting vs. non-law enacting states hovers near zero. This parallel trend during the pre-enactment period provides some evidence in support of meeting the parallel trends assumption.

References:

1. Roth, J., 2022. Pretest with caution: Event-study estimates after testing for parallel trends. *American Economic Review: Insights*, *4*(3), pp.305-322.

2. Sun L, Abraham S. Estimating dynamic treatment effects in event studies with heterogeneous treatment effects. Journal of econometrics. 2021 Dec 1;225(2):175-99.

3. https://cran.r-project.org/web/packages/fixest/vignettes/fixest_walkthrough.html#423_Staggered_difference-in-differences_(Sun_and_Abraham,_2020)

© 2025 Mannes ZL et al. *JAMA Health Forum*.

eTable 1. Event times (i.e. years prior and post passage) used in diff-in-diff estimation for each state for MCL

Event_times	States passing MCL during the period from 2005-2022
-17	MS
-16	AL, MS, SD
-15	AL, MS, SD, VA
-14	AL, MS, SD, VA
-13	AL, MO, MS, OK, SD, UT, VA
-12	AL, FL, MO, MS, OK, SD, UT, VA, WV
-11	AL, AR, FL, LA, MO, MS, ND, OH, OK, PA, SD, UT, VA, WV
-10	AL, AR, FL, LA, MO, MS, ND, OH, OK, PA, SD, UT, VA, WV
-9	AL, AR, FL, IL, LA, MN, MO, MS, ND, NY, OH, OK, PA, SD, UT, VA, WV
-8	AL, AR, FL, IL, LA, MA, MN, MO, MS, ND, NH, NY, OH, OK, PA, SD, UT, VA, WV
-7	AL, AR, CT, FL, IL, LA, MA, MN, MO, MS, ND, NH, NY, OH, OK, PA, SD, UT, VA, WV
-6	AL, AR, CT, DE, FL, IL, LA, MA, MN, MO, MS, ND, NH, NY, OH, OK, PA, SD, UT, VA, WV
-5	AL, AR, AZ, CT, DC, DE, FL, IL, LA, MA, MN, MO, MS, ND, NH, NJ, NY, OH, OK, PA, SD, UT, VA, WV
-4	AL, AR, AZ, CT, DC, DE, FL, IL, LA, MA, MN, MO, MS, ND, NH, NJ, NY, OH, OK, PA, SD, UT, VA, WV
-3	AL, AR, AZ, CT, DC, DE, FL, IL, LA, MA, MI, MN, MO, MS, ND, NH, NJ, NY, OH, OK, PA, SD, UT, VA, WV
-2	AL, AR, AZ, CT, DC, DE, FL, IL, LA, MA, MI, MN, MO, MS, ND, NH, NJ, NM, NY, OH, OK, PA, SD, UT, VA, WV
-1	AL, AR, AZ, CT, DC, DE, FL, IL, LA, MA, MI, MN, MO, MS, ND, NH, NJ, NM, NY, OH, OK, PA, RI, SD, UT, VA, WV
0	AL, AR, AZ, CT, DC, DE, FL, GA, IA, ID, IL, IN, KS, KY, LA, MA, MI, MN, MO, MS, NC, ND, NE, NH, NJ, NM, NY, OH, OK, PA, RI, SC, SD, TN, TX, UT, VA, WI, WV, WY
1	AL, AR, AZ, CT, DC, DE, FL, IL, LA, MA, MI, MN, MO, ND, NH, NJ, NM, NY, OH, OK, PA, RI, SD, UT, VA, WV
2	AR, AZ, CT, DC, DE, FL, IL, LA, MA, MI, MN, MO, ND, NH, NJ, NM, NY, OH, OK, PA, RI, UT, VA, WV
3	AR, AZ, CT, DC, DE, FL, IL, LA, MA, MI, MN, MO, ND, NH, NJ, NM, NY, OH, OK, PA, RI, UT, WV
4	AR, AZ, CT, DC, DE, FL, IL, LA, MA, MI, MN, MO, ND, NH, NJ, NM, NY, OH, OK, PA, RI, UT, WV
5	AR, AZ, CT, DC, DE, FL, IL, LA, MA, MI, MN, ND, NH, NJ, NM, NY, OH, PA, RI, WV
6	AR, AZ, CT, DC, DE, IL, LA, MA, MI, MN, ND, NH, NJ, NM, NY, OH, PA, RI
7	AZ, CT, DC, DE, IL, MA, MI, MN, NH, NJ, NM, NY, RI
8	AZ, CT, DC, DE, IL, MA, MI, MN, NH, NJ, NM, NY, RI
9	AZ, CT, DC, DE, MA, MI, NH, NJ, NM, RI
10	AZ, CT, DC, DE, MI, NJ, NM, RI
11	AZ, DC, DE, MI, NJ, NM, RI
12	AZ, DC, MI, NJ, NM, RI
13	MI, NM, RI

© 2025 Mannes ZL et al. *JAMA Health Forum*.

14	MI, NM, RI
15	NM, RI
16	RI

© 2025 Mannes ZL et al. *JAMA Health Forum*.

eTable 2. Event times (i.e. years prior and post passage) used in diff-in-diff estimation for each state for RCL

Event_times	States passing RCL during the period 2005 to 2022
-17	MO, RI
-16	CT, MO, MT, NJ, NM, NY, RI, VA
-15	AZ, CT, MO, MT, NJ, NM, NY, RI, VA
-14	AZ, CT, IL, MO, MT, NJ, NM, NY, RI, VA
-13	AZ, CT, IL, MI, MO, MT, NJ, NM, NY, RI, VA, VT
-12	AZ, CT, IL, ME, MI, MO, MT, NJ, NM, NV, NY, RI, VA, VT
-11	AZ, CA, CT, IL, MA, ME, MI, MO, MT, NJ, NM, NV, NY, RI, VA, VT
-10	AK, AZ, CA, CT, DC, IL, MA, ME, MI, MO, MT, NJ, NM, NV, NY, OR, RI, VA, VT
-9	AK, AZ, CA, CT, DC, IL, MA, ME, MI, MO, MT, NJ, NM, NV, NY, OR, RI, VA, VT
-8	AK, AZ, CA, CT, DC, IL, MA, ME, MI, MO, MT, NJ, NM, NV, NY, OR, RI, VA, VT
-7	AK, AZ, CA, CO, CT, DC, IL, MA, ME, MI, MO, MT, NJ, NM, NV, NY, OR, RI, VA, VT, WA
-6	AK, AZ, CA, CO, CT, DC, IL, MA, ME, MI, MO, MT, NJ, NM, NV, NY, OR, RI, VA, VT, WA
-5	AK, AZ, CA, CO, CT, DC, IL, MA, ME, MI, MO, MT, NJ, NM, NV, NY, OR, RI, VA, VT, WA
-4	AK, AZ, CA, CO, CT, DC, IL, MA, ME, MI, MO, MT, NJ, NM, NV, NY, OR, RI, VA, VT, WA
-3	AK, AZ, CA, CO, CT, DC, IL, MA, ME, MI, MO, MT, NJ, NM, NV, NY, OR, RI, VA, VT, WA
-2	AK, AZ, CA, CO, CT, DC, IL, MA, ME, MI, MO, MT, NJ, NM, NV, NY, OR, RI, VA, VT, WA
-1	AK, AZ, CA, CO, CT, DC, IL, MA, ME, MI, MO, MT, NJ, NM, NV, NY, OR, RI, VA, VT, WA
0	AK, AL, AR, AZ, CA, CO, CT, DC, DE, FL, GA, HI, IA, ID, IL, IN, KS, KY, LA, MA, MD, ME, MI, MN, MO, MS, MT, NC, ND, NE, NH, NJ, NM, NV, NY, OH, OK, OR, PA, RI, SC, SD, TN, TX, UT, VA, VT, WA, WI, WV, WY
1	AK, AZ, CA, CO, CT, DC, IL, MA, ME, MI, MT, NJ, NM, NV, NY, OR, VA, VT, WA
2	AK, AZ, CA, CO, DC, IL, MA, ME, MI, NV, OR, VT, WA
3	AK, CA, CO, DC, IL, MA, ME, MI, NV, OR, VT, WA
4	AK, CA, CO, DC, MA, ME, MI, NV, OR, VT, WA
5	AK, CA, CO, DC, MA, ME, NV, OR, WA
6	AK, CA, CO, DC, MA, OR, WA
7	AK, CO, DC, OR, WA
8	CO, WA
9	CO, WA
10	CO, WA

© 2025 Mannes ZL et al. *JAMA Health Forum*.

eFigure 1. Staggered adoption of when MCL and RCL were passed in all 50 states and Washington D.C.: 2005 to 2022

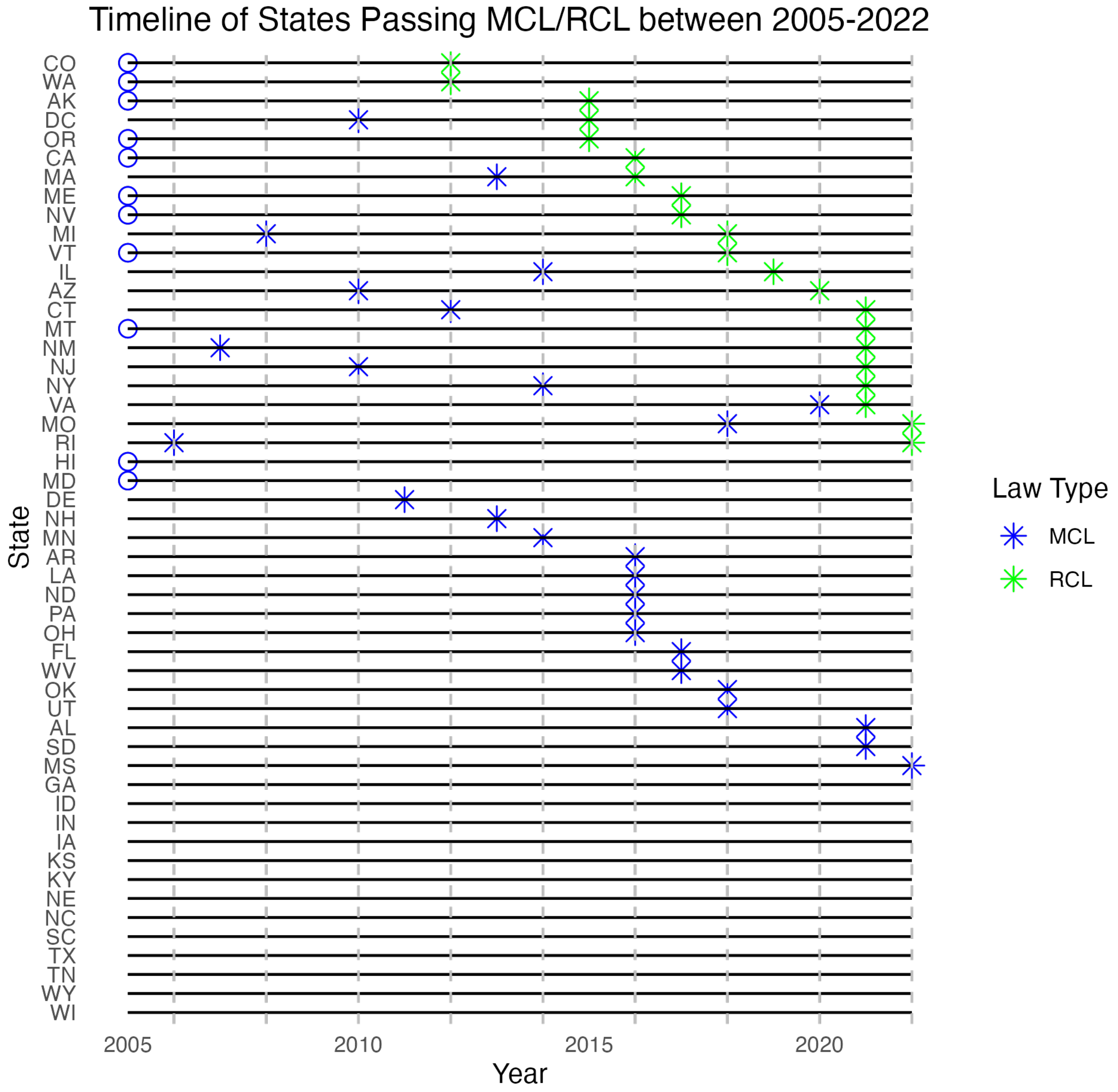

Note. Open blue circles on left indicate states with MCL prior to 2005. States at bottom of figure with no asterisks did not pass cannabis laws before 2022.

© 2025 Mannes ZL et al. *JAMA Health Forum*.

eFigure 2. Event study for OUD among VHA patients

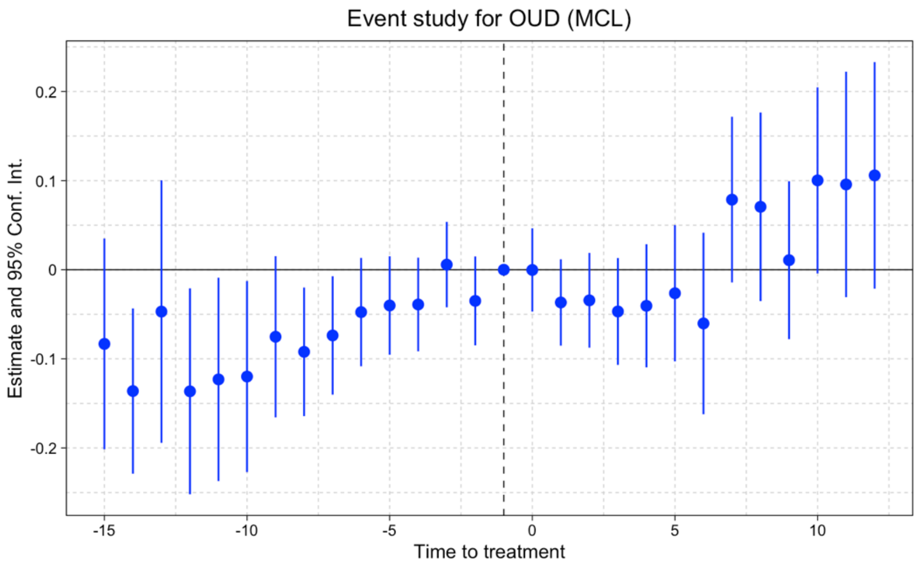

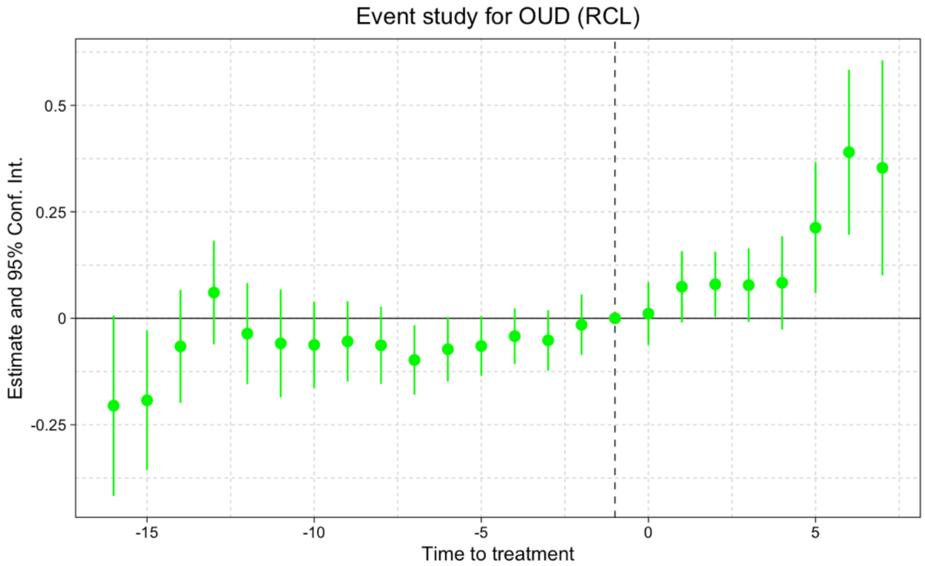

Note. Plot of differences in OUD between law states and non-law states prior to (negative) and post (positive) passage for MCL (top figure) and RCL (bottom figure). Estimates are from Sun & Abraham model for difference between OUD in states that passed laws and those that did not incorporating staggered adoption. See eTables 1 and 2 for lists of states contributing to each event time. Only event times with >3 states contributing are analyzed to avoid outlying values due to limited information. Use of event time -1 as a reference for pre vs post is commonly chosen rather than 0 since the effects of policy may already be present during the year 0 since that is the year the law is passed, and data is taken from that whole year. This would distort the pre-treatment and subsequent post-treatment comparisons.

© 2025 Mannes ZL et al. *JAMA Health Forum*.

eTable 3: Sample demographics by opioid use disorder status: 2005 and 2022

Characteristic	2005		2022	
	**OUD** N = 26,533[1]	**No OUD** N = 3,207,849[1]	**OUD** N = 49,923[1]	**No OUD** N = 4,386,960[1]
Age (continuous)	51.36 (8.21)	57.95 (12.16)	54.19 (13.67)	56.03 (14.66)
Age (categorical)				
<35	1,041 (3.9%)	181,768 (5.7%)	4,131 (8.3%)	464,598 (10.6%)
35-64	24,420 (92.0%)	1,969,072 (61.4%)	30,676 (61.4%)	2,307,924 (52.6%)
65-75	1,072 (4.0%)	1,057,009 (33.0%)	15,116 (30.3%)	1,614,438 (36.8%)
Sex				
Female	1,039 (3.9%)	190,107 (5.9%)	4,424 (8.9%)	582,155 (13.3%)
Male	25,494 (96.1%)	3,017,742 (94.1%)	45,499 (91.1%)	3,804,805 (86.7%)
Race/Ethnicity				
White	13,908 (52.4%)	2,412,064 (75.2%)	35,678 (71.5%)	2,733,388 (62.3%)
Black	9,888 (37.3%)	523,921 (16.3%)	8,904 (17.8%)	932,054 (21.2%)
Hispanic/Latino	1,649 (6.2%)	118,920 (3.7%)	2,822 (5.7%)	344,664 (7.9%)
Asian	42 (0.2%)	17,182 (0.5%)	183 (0.4%)	64,936 (1.5%)
AmInd/AlaskNative	136 (0.5%)	18,116 (0.6%)	460 (0.9%)	32,438 (0.7%)
PacIs/NatHawaiian	120 (0.5%)	22,753 (0.7%)	271 (0.5%)	34,977 (0.8%)
MultipleRace/Eth	227 (0.9%)	22,517 (0.7%)	469 (0.9%)	44,895 (1.0%)
Unknown	563 (2.1%)	72,376 (2.3%)	1,136 (2.3%)	199,608 (4.6%)

Abbreviations: OUD=opioid use disorder; AmInd/AlaskNative=American Indian or Alaskan Native; PacIs/NatHawaiian=Pacific Islander or Native Hawaiian
[a]Mean (SD)
[b]n (%)

© 2025 Mannes ZL et al. *JAMA Health Forum*.

eTable 4: Yearly overall prevalence of opioid use disorder

	OUD prevalence
Year	% (95% CI)
2005	1.12 (1.12, 1.13)
2006	1.15 (1.14, 1.15)
2007	1.11 (1.1, 1.12)
2008	1.12 (1.12, 1.13)
2009	1.11 (1.1, 1.11)
2010	1.09 (1.08, 1.09)
2011	1.09 (1.09, 1.1)
2012	1.11 (1.1, 1.11)
2013	1.12 (1.11, 1.12)
2014	1.13 (1.13, 1.14)
2015	1.14 (1.14, 1.15)
2016	1.23 (1.22, 1.23)
2017	1.25 (1.24, 1.25)
2018	1.23 (1.22, 1.24)
2019	1.22 (1.21, 1.23)
2020	1.21 (1.2, 1.22)
2021	1.17 (1.16, 1.18)
2022	1.12 (1.12, 1.13)

Abbreviations: OUD=opioid use disorder; CI=confidence interval

© 2025 Mannes ZL et al. *JAMA Health Forum*.

eTable 5: Yearly opioid use disorder prevalence by state cannabis law status, overall and by age groups

OUD prevalence: Overall			
Year	No CL<br>% (95% CI)	MCL<br>% (95% CI)	RCL<br>% (95% CI)
2005	1.12 (1.11, 1.12)	1.13 (1.12, 1.14)	1.13 (1.12, 1.14)
2006	1.16 (1.16, 1.17)	1.13 (1.12, 1.14)	1.15 (1.15, 1.16)
2007	1.10 (1.10, 1.11)	1.11 (1.1, 1.11)	1.12 (1.11, 1.12)
2008	1.12 (1.11, 1.12)	1.10 (1.09, 1.1)	1.15 (1.14, 1.16)
2009	1.11 (1.11, 1.12)	1.12 (1.12, 1.13)	1.09 (1.09, 1.1)
2010	1.09 (1.08, 1.09)	1.09 (1.08, 1.1)	1.08 (1.07, 1.09)
2011	1.06 (1.06, 1.07)	1.14 (1.13, 1.15)	1.08 (1.07, 1.09)
2012	1.05 (1.05, 1.06)	1.13 (1.12, 1.14)	1.13 (1.12, 1.14)
2013	1.05 (1.05, 1.06)	1.15 (1.13, 1.16)	1.14 (1.13, 1.15)
2014	1.08 (1.07, 1.08)	1.16 (1.15, 1.17)	1.16 (1.14, 1.17)
2015	1.09 (1.09, 1.1)	1.18 (1.17, 1.19)	1.16 (1.15, 1.17)
2016	1.15 (1.14, 1.16)	1.28 (1.27, 1.29)	1.25 (1.23, 1.26)
2017	1.17 (1.16, 1.18)	1.29 (1.27, 1.3)	1.28 (1.26, 1.29)
2018	1.19 (1.18, 1.2)	1.27 (1.26, 1.29)	1.23 (1.22, 1.24)
2019	1.14 (1.13, 1.15)	1.28 (1.27, 1.3)	1.23 (1.22, 1.24)
2020	1.13 (1.12, 1.14)	1.27 (1.25, 1.28)	1.22 (1.21, 1.24)
2021	1.10 (1.09, 1.11)	1.23 (1.22, 1.24)	1.17 (1.16, 1.18)
2022	1.06 (1.05, 1.07)	1.19 (1.17, 1.2)	1.13 (1.11, 1.14)
**OUD prevalence: 18-34**			
**Year**	**No CL<br>% (95% CI)**	**MCL<br>% (95% CI)**	**RCL<br>% (95% CI)**
2005	1.54 (1.51, 1.56)	1.71 (1.68, 1.75)	1.61 (1.59, 1.64)
2006	1.65 (1.63, 1.67)	1.73 (1.7, 1.76)	1.78 (1.76, 1.81)
2007	1.7 (1.68, 1.73)	1.76 (1.73, 1.78)	1.75 (1.73, 1.77)
2008	1.7 (1.68, 1.72)	1.56 (1.53, 1.59)	1.76 (1.74, 1.78)
2009	1.58 (1.56, 1.6)	1.58 (1.54, 1.61)	1.65 (1.63, 1.68)
2010	1.63 (1.61, 1.65)	1.67 (1.63, 1.7)	1.62 (1.61, 1.64)
2011	1.62 (1.61, 1.64)	1.7 (1.64, 1.75)	1.61 (1.58, 1.63)
2012	1.69 (1.67, 1.71)	1.77 (1.72, 1.82)	1.63 (1.6, 1.66)
2013	1.62 (1.59, 1.64)	1.87 (1.81, 1.93)	1.63 (1.6, 1.67)
2014	1.6 (1.57, 1.62)	1.84 (1.78, 1.89)	1.65 (1.61, 1.69)
2015	1.68 (1.67, 1.7)	1.83 (1.78, 1.89)	1.63 (1.59, 1.66)
2016	1.71 (1.69, 1.73)	1.97 (1.91, 2.02)	1.69 (1.65, 1.73)
2017	1.67 (1.64, 1.71)	1.88 (1.82, 1.93)	1.66 (1.63, 1.7)
2018	1.67 (1.64, 1.7)	1.77 (1.72, 1.83)	1.53 (1.5, 1.57)
2019	1.67 (1.65, 1.69)	1.66 (1.61, 1.71)	1.52 (1.49, 1.55)
2020	1.56 (1.54, 1.57)	1.57 (1.53, 1.62)	1.47 (1.44, 1.5)
2021	1.55 (1.54, 1.57)	1.55 (1.53, 1.57)	1.41 (1.38, 1.43)
2022	1.47 (1.45, 1.49)	1.46 (1.43, 1.49)	1.40 (1.37, 1.43)
**OUD prevalence: 35-64**			
**Year**	**No CL<br>% (95% CI)**	**MCL<br>% (95% CI)**	**RCL<br>% (95% CI)**
2005	0.49 (0.47, 0.5)	0.73 (0.71, 0.75)	0.93 (0.91, 0.95)
2006	0.52 (0.51, 0.54)	0.77 (0.75, 0.79)	0.96 (0.94, 0.98)
2007	0.51 (0.49, 0.53)	0.72 (0.7, 0.73)	0.9 (0.89, 0.92)
2008	0.51 (0.49, 0.52)	0.68 (0.66, 0.69)	0.85 (0.84, 0.87)
2009	0.45 (0.44, 0.47)	0.6 (0.59, 0.61)	0.72 (0.7, 0.73)
2010	0.48 (0.47, 0.5)	0.66 (0.64, 0.67)	0.73 (0.72, 0.75)
2011	0.48 (0.47, 0.49)	0.67 (0.65, 0.68)	0.7 (0.69, 0.71)
2012	0.55 (0.54, 0.57)	0.71 (0.69, 0.72)	0.72 (0.71, 0.74)
2013	0.55 (0.54, 0.57)	0.76 (0.75, 0.78)	0.74 (0.72, 0.75)
2014	0.61 (0.6, 0.62)	0.81 (0.8, 0.83)	0.76 (0.74, 0.77)
2015	0.69 (0.68, 0.71)	0.89 (0.87, 0.9)	0.8 (0.79, 0.82)
2016	0.86 (0.84, 0.88)	1.09 (1.07, 1.11)	0.93 (0.91, 0.95)

© 2025 Mannes ZL et al. *JAMA Health Forum*.

2017	0.9 (0.88, 0.92)	1.12 (1.1, 1.14)	0.92 (0.9, 0.94)
2018	0.87 (0.85, 0.89)	1.04 (1.02, 1.06)	0.83 (0.81, 0.84)
2019	0.78 (0.76, 0.8)	1 (0.98, 1.02)	0.76 (0.74, 0.77)
2020	0.71 (0.7, 0.73)	0.93 (0.91, 0.95)	0.68 (0.66, 0.69)
2021	0.66 (0.64, 0.68)	0.85 (0.83, 0.87)	0.59 (0.58, 0.61)
2022	0.56 (0.55, 0.58)	0.71 (0.69, 0.73)	0.47 (0.46, 0.49)

OUD prevalence: 65-75			
**Year**	**No CL**	**MCL**	**RCL**
	% (95% CI)	% (95% CI)	% (95% CI)
2005	0.45 (0.44, 0.46)	0.45 (0.45, 0.46)	0.45 (0.44, 0.45)
2006	0.47 (0.46, 0.47)	0.45 (0.45, 0.46)	0.44 (0.43, 0.45)
2007	0.46 (0.46, 0.47)	0.45 (0.45, 0.46)	0.43 (0.43, 0.44)
2008	0.44 (0.44, 0.45)	0.45 (0.44, 0.45)	0.42 (0.41, 0.42)
2009	0.43 (0.42, 0.43)	0.43 (0.42, 0.43)	0.42 (0.41, 0.42)
2010	0.44 (0.43, 0.44)	0.44 (0.43, 0.45)	0.42 (0.42, 0.43)
2011	0.43 (0.43, 0.44)	0.43 (0.42, 0.43)	0.42 (0.42, 0.43)
2012	0.42 (0.42, 0.43)	0.43 (0.43, 0.44)	0.43 (0.42, 0.44)
2013	0.43 (0.42, 0.43)	0.44 (0.43, 0.45)	0.45 (0.44, 0.46)
2014	0.43 (0.42, 0.43)	0.45 (0.44, 0.46)	0.48 (0.47, 0.49)
2015	0.45 (0.44, 0.46)	0.47 (0.46, 0.48)	0.49 (0.48, 0.5)
2016	0.49 (0.48, 0.5)	0.52 (0.51, 0.54)	0.57 (0.55, 0.58)
2017	0.52 (0.5, 0.53)	0.56 (0.55, 0.57)	0.64 (0.63, 0.66)
2018	0.55 (0.53, 0.56)	0.61 (0.59, 0.62)	0.67 (0.66, 0.69)
2019	0.56 (0.55, 0.57)	0.66 (0.64, 0.67)	0.73 (0.71, 0.74)
2020	0.58 (0.56, 0.59)	0.67 (0.65, 0.68)	0.77 (0.75, 0.79)
2021	0.59 (0.57, 0.6)	0.68 (0.66, 0.7)	0.79 (0.77, 0.81)
2022	0.61 (0.6, 0.63)	0.72 (0.7, 0.74)	0.86 (0.83, 0.88)

Abbreviations: OUD=opioid use disorder; CL=cannabis law; MCL=medical cannabis law; RCL=recreational cannabis law; CI=confidence interval

© 2025 Mannes ZL et al. *JAMA Health Forum*.

eTable 6. Adjusted OUD prevalence in Veterans Health Administration patients with versus without chronic pain in 2005 and 2022, by MCL/RCL, by age group

	18-34 Years			35-64 Years			65-75 Years		
	OUD prevalence[e]		Absolute Change	OUD prevalence[e]		Absolute Change	OUD prevalence[e]		Absolute Change
**Chronic Pain**									
**Type of State**	2005 N=60,101	2022 N=187,389	%	2005 N=868,355	2022 N=1,224,448	%	2005 N=353,565	2022 N=828,245	%
No CL[a]	2.21	2.36	0.16	2.02	1.79	-0.23	0.77	1.06	0.30
MCL[b]	2.36	2.30	-0.06	1.96	1.99	0.03	0.76	1.24	0.48
RCL[c]	2.21	2.22	0.01	2.05	1.79	-0.26	0.75	1.44	0.70
**No Chronic Pain**									
**Type of State**	2005 N=122,708	2022 N=281,340	%	2005 N=1,125,137	2022 N=1,114,152	%	2005 N=704,516	2022 N=801,309	%
No CL[a]	0.89	0.93	0.05	0.91	0.64	-0.27	0.22	0.21	-0.01
MCL[b]	1.24	0.88	-0.35	0.91	0.81	-0.11	0.21	0.24	0.03
RCL[c]	1.15	0.92	-0.23	1.09	0.60	-0.49	0.21	0.30	0.08

Abbreviations: OUD=opioid use disorder; CL=cannabis law; MCL=medical cannabis law; RCL=recreational cannabis law

[a] 13 states

[b] 17 states

[c] 20 states and Washington D.C.

[d] adjusted for categorical age, sex, race/ethnicity, mandatory PDMP access law, receipt of ≥30-days of prescription opioids, other substance use disorder (alcohol use disorder, cocaine use disorder, stimulant use disorder, sedative use disorder, hallucinogen related disorders, inhalant related disorders other psychoactive substance related disorder), and time-varying state covariates including yearly state-level median income, yearly state rates of male individuals, Hispanic individuals, non-Hispanic Black individuals, non-Hispanic White individuals, those under federal poverty level, those aged ≥18, those who are unemployed.

[e] adjusted for continuous age, sex, race/ethnicity, mandatory PDMP access law, receipt of ≥30-days of prescription opioids, other substance use disorder (alcohol use disorder, cocaine use disorder, stimulant use disorder, sedative use disorder, hallucinogen related disorders, inhalant related disorders other psychoactive substance related disorder), and time-varying state covariates including yearly state-level median income, yearly state rates of male individuals, Hispanic individuals, non-Hispanic Black individuals, non-Hispanic White individuals, those under federal poverty level, those aged ≥18, those who are unemployed.

© 2025 Mannes ZL et al. *JAMA Health Forum*.

eTable 7: State MCL and RCL enactment and opioid use disorder prevalence in Veterans Health Administration patients with versus without chronic pain, overall and by age group

		Overall		18-34 Years		35-64 Years		65-75 Years	
	Change in state law[a]	DiD law result (95% CI)[b]	p	DiD law result (95% CI)[d]	p	DiD law result (95% CI)[d]	p	DiD law result (95% CI)[d]	p
**Chronic Pain**									
	**No CL to MCL**	0.078 (0.068, 0.088)	<.001	0.070 (0.036, 0.105)	<.001	0.090 (0.075, 0.106)	<.001	0.056 (0.044, 0.069)	<.001
	**MCL to RCL**	0.134 (0.120, 0.149)	<.001	-0.010 (-0.048, 0.028)	0.597	0.091 (0.069, 0.113)	<.001	0.230 (0.207, 0.253)	<.001
**No Chronic Pain**									
	**No CL to MCL**	0.025 (0.019, 0.031)	<.001	-0.007 (-0.023, 0.016)	0.558	0.015 (0.004, 0.026)	0.006	0.016 (0.010, 0.022)	<.001
	**MCL to RCL**	0.009 (0.001, 0.018)	0.025	-0.011 (-0.038, 0.015)	0.400	-0.005 (-0.019, 0.008)	0.449	0.029 (0.020, 0.039)	<.001

Abbreviations: CL=cannabis law; MCL=medical cannabis law; RCL=recreational cannabis law; DiD: difference-in-difference

[a] From 2005-2022, 26 states and Washington, DC enacted MCL-only from 2005 to 2022 and 11 states and Washington, DC transitioned from MCL-only to RCL/MCL. Three states and Washington, DC, made both changes between 2005 and 2022 (i.e., no CL to MCL only and then later to RCL/MCL), and therefore contributed data to both associations. There were 15 states (2 with MCLs only and 13 with no CLs in 2022) that made no law changes between 2005 and 2022; in the DiD model, they contribute to background secular trends. Model estimated effects represent the absolute increase or decrease in OUD prevalence associated with law enactment. The DiD model compares the years after enactment (up to 2022 or until the next law change) in each state to the years before enactment (since 2005 or the previous law change) in the same state and controls for contemporaneous trends in other states that have not yet passed the respective law.

[b] Absolute % change in prevalence of OUD.. Adjusted for categorical age, sex, race and ethnicity, mandatory PDMP access law, 30-day opioid prescription, other SUD, and time-varying state covariates including yearly state-level median income, and yearly state rates of male individuals, Hispanic individuals, non-Hispanic Black individuals, non-Hispanic White individuals, those in the poverty category, those 18 years and older, and those who are unemployed.

[c] DiD estimate divided by absolute change in OUD prevalence.

[d] Adjusted for continuous age, sex, race/ethnicity, mandatory PDMP access law, receipt of ≥30-days of prescription opioids, other substance use disorder (alcohol use disorder, cocaine use disorder, stimulant use disorder, sedative use disorder, hallucinogen related disorders, inhalant related disorders other psychoactive substance related disorder), and time-varying state covariates including yearly state-level median income, yearly state rates of male individuals, Hispanic individuals, non-Hispanic Black individuals, non-Hispanic White individuals, those under federal poverty level, those aged ≥18, those who are unemployed.

© 2025 Mannes ZL et al. *JAMA Health Forum*.

eTable 8. State dispensary enactment and opioid use disorder prevalence in Veterans Health Administration patients, overall and by age group

	Overall		18-34 Years		35-64 Years		65-75 Years	
Dispensary Status[a]	DiD law result (95% CI)[b]	p	DiD law result (95% CI)[d]	p	DiD law result (95% CI)[d]	p	DiD law result (95% CI)[d]	p
**Effect of change to medical dispensaries**	0.071 (0.065, 0.076)	<0.001	0.011 (-0.006, 0.029)	0.197	0.073 (0.064, 0.083)	<0.001	0.056 (0.049, 0.63)	<0.001
**Effect of change to recreational dispensaries**	0.052 (0.042, 0.061)	<0.001	-0.009 (-0.032, 0.014)	0.420	0.035 (0.020, 0.050)	<0.001	0.102 (0.088, 0.115)	<0.001

Abbreviations: DiD: difference-in-difference

[a] From 2005-2022, 19 states and DC made a change to having medical dispensaries during the period; 1 states made a change to having recreational dispensaries and 16 states made a change to medical and to recreational dispensaries; in the DiD model, they contribute to background secular trends. Model estimated effects represent the absolute increase or decrease in OUD prevalence associated with law enactment. The DiD model compares the years after enactment (up to 2022 or until the next law change) in each state to the years before enactment (since 2005 or the previous law change) in the same state and controls for contemporaneous trends in other states that have not yet passed the respective law.

[b] Absolute % change in prevalence of OUD. . Adjusted for categorical age, sex, race and ethnicity, mandatory PDMP access law, receipt of ≥30-days of prescription opioids, other substance use disorder (alcohol use disorder, cocaine use disorder, stimulant use disorder, sedative use disorder, hallucinogen related disorders, inhalant related disorders other psychoactive substance related disorder), and time-varying state covariates including yearly state-level median income, and yearly state rates of male individuals, Hispanic individuals, non-Hispanic Black individuals, non-Hispanic White individuals, those in the poverty category, those 18 years and older, and those who are unemployed.

[c] DiD estimate divided by absolute change in OUD prevalence.

[d] Adjusted for continuous age, sex, race/ethnicity, mandatory PDMP access law, receipt of ≥30-days of prescription opioids, other substance use disorder (alcohol use disorder, cocaine use disorder, stimulant use disorder, sedative use disorder, hallucinogen related disorders, inhalant related disorders other psychoactive substance related disorder), and time-varying state covariates including yearly state-level median income, yearly state rates of male individuals, Hispanic individuals, non-Hispanic Black individuals, non-Hispanic White individuals, those under federal poverty level, those aged ≥18, those who are unemployed.

© 2025 Mannes ZL et al. *JAMA Health Forum*.

eTable 9. State dispensary status and opioid use disorder prevalence in Veterans Health Administration patients with versus without chronic pain, overall and by age group

		Overall		18-34 Years		35-64 Years		65-75 Years	
	Dispensary Status[a]	DiD law result (95% CI)[b]	p	DiD law result (95% CI)[d]	p	DiD law result (95% CI)[d]	p	DiD law result (95% CI)[d]	p
**Chronic Pain**									
	**Effect of change to medical dispensaries**	0.105 (0.095, 0.115)	<.001	0.032 (0.000, 0.064)	0.047	0.117 (0.102, 0.133)	<.001	0.099 (0.085, 0.113)	<.001
	**Effect of change to recreational dispensaries**	0.109 (0.093, 0.125)	<.001	0.019 (-0.021, 0.058)	0.357	0.077 (0.052, 0.102)	<.001	0.198 (0.172, 0.224)	<.001
**No Chronic Pain**									
	**Effect of change to medical dispensaries**	0.025 (0.020, 0.031)	<.001	0.004 (-0.017, 0.026)	0.702	0.024 (0.014, 0.035)	<.001	0.017 (0.011, 0.024)	<.001
	**Effect of change to recreational dispensaries**	-0.002 (-0.011, 0.008)	0.745	0.012 (-0.017, 0.040)	0.428	-0.010 (-0.027, 0.007)	0.251	0.017 (0.006, 0.028)	0.003

Abbreviations: DiD: difference-in-difference

[a] From 2005-2022, 19 states and DC made a change to having medical dispensaries during the period; 1 states made a change to having recreational dispensaries and 16 states made a change to medical and to recreational dispensaries; in the DiD model, they contribute to background secular trends. Model estimated effects represent the absolute increase or decrease in OUD prevalence associated with law enactment. The DiD model compares the years after enactment (up to 2022 or until the next law change) in each state to the years before enactment (since 2005 or the previous law change) in the same state and controls for contemporaneous trends in other states that have not yet passed the respective law.

[b] Absolute % change in prevalence of OUD. Adjusted for categorical age, sex, race and ethnicity, mandatory PDMP access law, 30-day opioid prescription, other SUD, and time-varying state covariates including yearly state-level median income, and yearly state rates of male individuals, Hispanic individuals, non-Hispanic Black individuals, non-Hispanic White individuals, those in the poverty category, those 18 years and older, and those who are unemployed.

[c] DiD estimate divided by absolute change in OUD prevalence.

[d] Adjusted for continuous age, sex, race/ethnicity, mandatory PDMP access law, receipt of ≥30-days of prescription opioids, other substance use disorder (alcohol use disorder, cocaine use disorder, stimulant use disorder, sedative use disorder, hallucinogen related disorders, inhalant related disorders other psychoactive substance related disorder), and time-varying state covariates including yearly state-level median income, yearly state rates of male individuals, Hispanic individuals, non-Hispanic Black individuals, non-Hispanic White individuals, those under federal poverty level, those aged ≥18, those who are unemployed.

© 2025 Mannes ZL et al. *JAMA Health Forum*.

eTable 10. State 1-year lag of MCL and RCL enactment and opioid use disorder prevalence in Veterans Health Administration patients, overall and by age group

	Overall		18-34 Years		35-64 Years		65-75 Years	
**Change in state law[a]**	**DiD law result (95% CI)[b]**	**p**	**DiD law result (95% CI)[d]**	**p**	**DiD law result (95% CI)[d]**	**p**	**DiD law result (95% CI)[d]**	**p**
**No CL to MCL**	0.006 (0.054, 0.066)	<0.001	0.013 (-0.005, 0.031)	0.150	0.051 (0.041, 0.061)	<0.001	0.042 (0.035, 0.49)	<0.001
**MCL to RCL**	0.075 (0.066, 0.083)	<0.001	-0.007 (-0.030, 0.016)	0.542	0.047 (0.033, 0.061)	<0.001	0.121 (0.109, 0.133)	<0.001

Abbreviations: CL=cannabis law; MCL=medical cannabis law; RCL=recreational cannabis law; DiD: difference-in-difference

[a] From 2005-2022, 26 states and DC made a change from no-CL to MCL-only during the period from 2005-2021 (to account for the 1-year lag); 19 states and DC made a change from MCL-only to RCL/MCL during the period. Note, 10 of these states and DC made both changes during the period from no-CL to MCL-only and then to RCL/MCL hence contribute to both effects. With the 1-year lag, 16 states (2 with MCL-only and 14 with no-CL) made no law changes between 2005-2022 and contribute in the DID model to background secular trends. Model estimated effects represent the absolute increase or decrease in OUD prevalence associated with law enactment. The DiD model compares the years after enactment (up to 2022 or until the next law change) in each state to the years before enactment (since 2005 or the previous law change) in the same state and controls for contemporaneous trends in other states that have not yet passed the respective law.

[b] Absolute % change in prevalence of OUD.. Adjusted for categorical age, sex, race and ethnicity, mandatory PDMP access law, receipt of ≥30-days of prescription opioids, other substance use disorder (alcohol use disorder, cocaine use disorder, stimulant use disorder, sedative use disorder, hallucinogen related disorders, inhalant related disorders other psychoactive substance related disorder), and time-varying state covariates including yearly state-level median income, and yearly state rates of male individuals, Hispanic individuals, non-Hispanic Black individuals, non-Hispanic White individuals, those in the poverty category, those 18 years and older, and those who are unemployed.

[c] DiD estimate divided by absolute change in OUD prevalence.

[d] Adjusted for continuous age, sex, race/ethnicity, mandatory PDMP access law, receipt of ≥30-days of prescription opioids, other substance use disorder (alcohol use disorder, cocaine use disorder, stimulant use disorder, sedative use disorder, hallucinogen related disorders, inhalant related disorders other psychoactive substance related disorder), and time-varying state covariates including yearly state-level median income, yearly state rates of male individuals, Hispanic individuals, non-Hispanic Black individuals, non-Hispanic White individuals, those under federal poverty level, those aged ≥18, those who are unemployed.

© 2025 Mannes ZL et al. *JAMA Health Forum*.

eTable 11: State 1-year lag MCL and RCL enactment and opioid use disorder prevalence in Veterans Health Administration patients with versus without chronic pain, overall and by age group

		Overall		18-34 Years		35-64 Years		65-75 Years	
	Change in state law[a]	DiD law result (95% CI)[b]	p	DiD law result (95% CI)[d]	p	DiD law result (95% CI)[d]	p	DiD law result (95% CI)[d]	p
**Chronic Pain**									
	**No CL to MCL**	0.078 (0.067, 0.088)	<.001	0.053 (0.020, 0.085)	0.002	0.087 (0.070, 0.103)	<.001	0.066 (0.052, 0.079)	<.001
	**MCL to RCL**	0.130 (0.114, 0.146)	<.001	0.013 (-0.026, 0.052)	0.513	0.096 (0.072, 0.119)	<.001	0.221 (0.196, 0.246)	<.001
**No Chronic Pain**									
	**No CL to MCL**	0.031 (0.025, 0.038)	<.001	0.001 (-0.022, 0.025)	0.915	0.020 (0.008, 0.031)	<.001	0.022 (0.015, 0.028)	<.001
	**MCL to RCL**	0.011 (0.002, 0.002)	0.013	0.012 (-0.016, 0.040)	0.410	-0.004 (-0.020, 0.012)	0.641	0.030 (0.019, 0.041)	<.001

Abbreviations: CL=cannabis law; MCL=medical cannabis law; RCL=recreational cannabis law; DiD: difference-in-difference

[a] From 2005-2022, 26 states and DC made a change from no-CL to MCL-only during the period from 2005-2021 (to account for the 1-year lag); 19 states and DC made a change from MCL-only to RCL/MCL during the period. Note, 10 of these states and DC made both changes during the period from no-CL to MCL-only and then to RCL/MCL hence contribute to both effects. With the 1-year lag, 16 states (2 with MCL-only and 14 with no-CL) made no law changes between 2005-2022 and contribute in the DID model to background secular trend. Model estimated effects represent the absolute increase or decrease in OUD prevalence associated with law enactment. The DiD model compares the years after enactment (up to 2022 or until the next law change) in each state to the years before enactment (since 2005 or the previous law change) in the same state and controls for contemporaneous trends in other states that have not yet passed the respective law.

[b] Absolute % change in prevalence of OUD.. Adjusted for categorical age, sex, race and ethnicity, mandatory PDMP access law, 30-day opioid prescription, other SUD, and time-varying state covariates including yearly state-level median income, and yearly state rates of male individuals, Hispanic individuals, non-Hispanic Black individuals, non-Hispanic White individuals, those in the poverty category, those 18 years and older, and those who are unemployed.

[c] DiD estimate divided by absolute change in OUD prevalence.

[d] Adjusted for continuous age, sex, race/ethnicity, mandatory PDMP access law, receipt of ≥30-days of prescription opioids, other substance use disorder (alcohol use disorder, cocaine use disorder, stimulant use disorder, sedative use disorder, hallucinogen related disorders, inhalant related disorders other psychoactive substance related disorder), and time-varying state covariates including yearly state-level median income, yearly state rates of male individuals, Hispanic individuals, non-Hispanic Black individuals, non-Hispanic White individuals, those under federal poverty level, those aged ≥18, those who are unemployed.

© 2025 Mannes ZL et al. *JAMA Health Forum*.

Appendix. ICD-9-CM and ICD-10-CM Codes for Substance Use Disorders

Substance Use Disorders	ICD-9-CM	ICD-10-CM
Alcohol use disorder	303.9X, 305.0X	F10.1X, F10.2X
Cocaine use disorder	304.2X, 305.6X	F14.1X, F14.2X
Stimulant use disorder	304.4X, 305.7X	F15.1X, F15.2X
Sedative Use Disorder	304.1X, 305.4X	F13.1X, F13.2X
Other substance use disorder[a]	304.5X, 305.3X, 304.6X, 304.8X, 304.9X, 305.8X, 305.9X	F19.1X, F19.2X, F16.1X, F16.2X, F18.1X, F18.2X

[a] Hallucinogen, inhalant, and other psychoactive substance related disorder

© 2025 Mannes ZL et al. *JAMA Health Forum*.
