# Supplementary material for: Cannabis Legalization and Opioid Use Disorder in Veterans Health Administration Patients
Source: JAMA Health Forum. 2025 Jun 13;6(6):e251369. doi: 10.1001/jamahealthforum.2025.1369 (PMC12166489; doi:10.1001/jamahealthforum.2025.1369)
Supplement: Supplement 2. — Data sharing statement [file jamahealthforum-e251369-s002.pdf]

Data Sharing Statement

Mannes. Cannabis Legalization and Opioid Use Disorder in Veterans Health Administration Patients. *JAMA Health Forum*. Published June 13, 2025.
doi:10.1001/jamahealthforum.2025.1369

Data
Data available: No
